# The Role of Cathelicidin LL-37 in Cancer Development

**DOI:** 10.1007/s00005-015-0359-5

**Published:** 2015-09-22

**Authors:** Ewelina Piktel, Katarzyna Niemirowicz, Urszula Wnorowska, Marzena Wątek, Tomasz Wollny, Katarzyna Głuszek, Stanisław Góźdź, Ilya Levental, Robert Bucki

**Affiliations:** Department of Microbiological and Nanobiomedical Engineering, Medical University of Białystok, Mickiewicza 2c, 15-222 Białystok, Poland; Holy Cross Oncology Center of Kielce, Kielce, Poland; The Faculty of Health Sciences of The Jan Kochanowski University in Kielce, Kielce, Poland; Department of Integrative Biology and Pharmacology, The University of Texas Medical School, Houston, TX USA; Department of Physiology, Pathophysiology and Microbiology of Infections, Faculty of Health Sciences of The Jan Kochanowski University in Kielce, Kielce, Poland

**Keywords:** Cancer, Immune system, Carcinogenesis, Cathelicidin, LL-37

## Abstract

LL-37 is a C-terminal peptide proteolytically released from 18 kDa human cathelicidin protein (hCAP18). Chronic infections, inflammation, tissue injury and tissue regeneration are all linked with neoplastic growth, and involve LL-37 antibacterial and immunomodulatory functions. Such a link points to the possible involvement of LL-37 peptide in carcinogenesis. An increasing amount of evidence suggests that LL-37 can have two different and contradictory effects—promotion or inhibition of tumor growth. The mechanisms are tissue-specific, complex, and depend mostly on the ability of LL-37 to act as a ligand for different membrane receptors whose expression varies on different cancer cells. Overexpression of LL-37 was found to promote development and progression of ovarian, lung and breast cancers, and to suppress tumorigenesis in colon and gastric cancer. This review explores and summarizes the current views on how LL-37 contributes to immunity, pathophysiology and cell signaling involved in malignant tumor growth.

## Introduction

Effective cancer treatment is one of the major challenges for modern medicine. It has been proven that the majority of these cases are strongly associated with the western lifestyle and only 5–10 % of the malignancies are genetically conditioned (Anand et al. 2008). Although present knowledge about risk factors increasing the possibility of cancer development is satisfactory, data of the mechanisms that directly cause malignant transformation are still insufficient. For this reason, increasing knowledge of the mechanisms governing the biology of the tumor should be a priority. We believe that particularly important issue should be searching of new links between cancer and the human immune system. Such a link represents a family of proteins named cathelicidins. They are essential components of innate immunity—together with defensins and other antimicrobial peptides (AMPs) they provide a first line of defense against a variety of pathogens. About 30 cathelicidin family members have been described in mammals, however, in humans only one, hCAP-18, has been identified. hCAP-18 is mostly expressed by neutrophils, monocytes, mast and dendritic cells (Agerberth et al. 2000; Vandamme et al. 2012), although its expression can also be induced during infection in epithelial cells and human keratinocytes (Bals et al. 1998; Frohm et al. 1997). hCAP-18 is a precursor for the AMP LL-37, which is released by proteinase 3-mediated extracellular cleavage (Scott et al. 2002; Sørensen et al. 2001). Many studies have revealed that hCAP-18 and LL-37 possess a wide range of pleiotropic properties (Bucki et al. 2010) including antimicrobial activities against bacteria, viruses, fungi and parasites (Bals et al. 1998; Barlow et al. 2011; Bucki et al. 2004; Bucki and Janmey 2006; Currie et al. 2013; Leszczynska et al. 2013; López-García et al. 2005; Rico-Mata et al. 2013; Wang 2014; Zaiou and Gallo 2002). At low concentrations, LL-37 inhibits the formation of bacterial biofilms, even in the case of microorganisms resistant to conventional antibiotics (Dosler and Karaaslan 2014; Overhage et al. 2008). However, the antimicrobial properties of LL-37 are reduced in patients with cystic fibrosis as a result of the interaction of the peptide with the DNA, F-actin and mucins (Bucki et al. 2007) or are inactivated by protease (Sieprawska-Lupa et al. 2004). In contrast to that, synthetic derivatives of AMPs are characterized by resistance to inactivation by polyelectrolytes (Bucki et al. 2007, 2008) and to protease digestion (Kuroda et al. 2013).

**Fig. 1:**
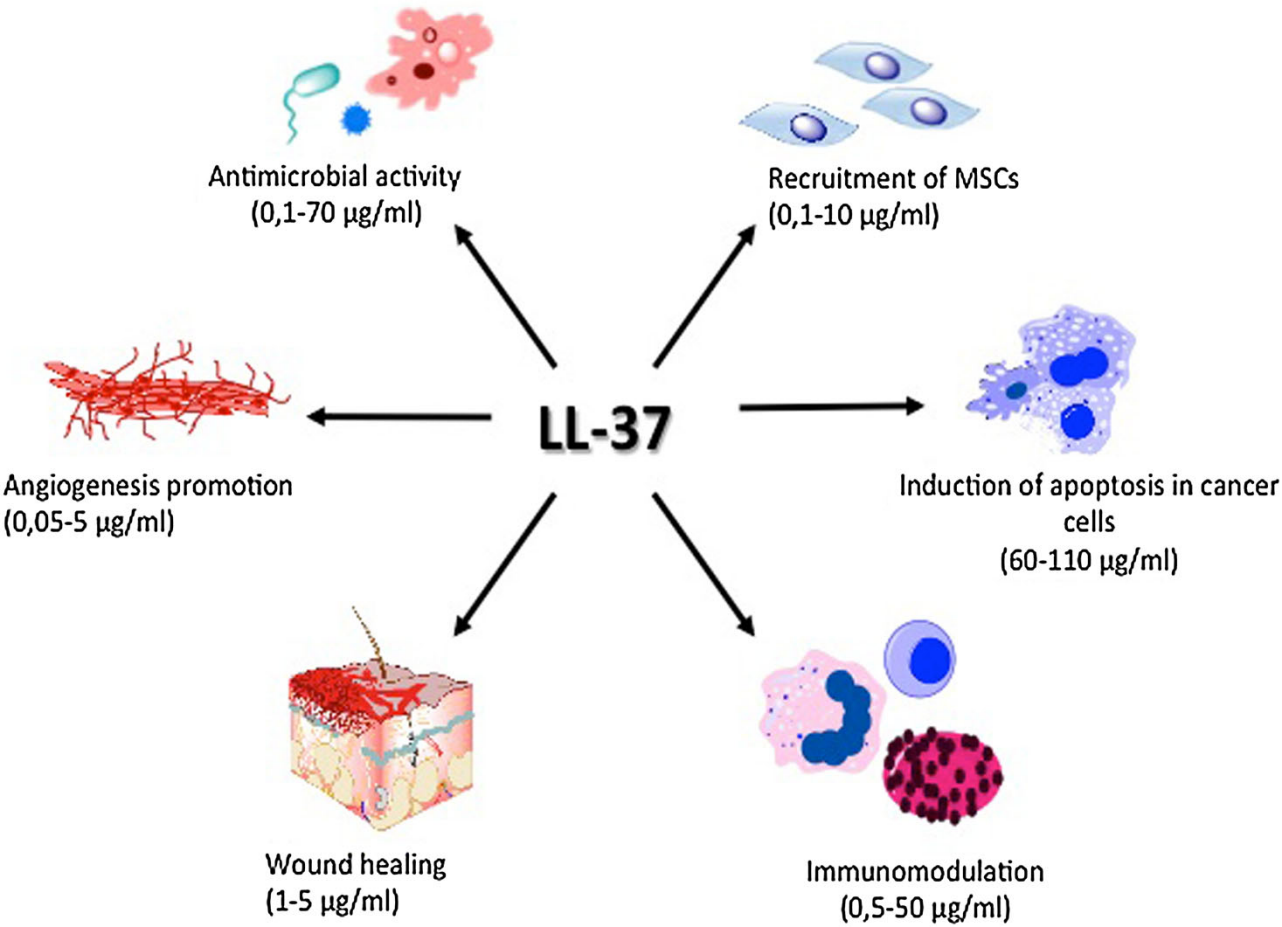
The pleiotropic properties of LL-37 in relation to the different cells and tissues. *MSCs:* mesenchymal stromal cells

The spectrum of LL-37 activity includes also activation of cell proliferation, epithelial cells migration, and promotion of wound closure, which together play an important role in tissue homeostasis and regenerative processes (Shaykhiev et al. 2005). A summary of the pleiotropic properties of the LL-37 peptide is presented in Fig. 1. Aside from its effect on individual cells, LL-37 may play an important role in governing the function of mucosal barriers, which implicates its more complex regulatory effect at tissues level. LL-37 treatment increases the stiffness and decreased the transepithelial permeability of confluent monolayer of lung epithelial cells, which correlated with decreased bacterial translocation into the cells (Byfield et al. 2011). Such effects are believed to prevent chronic infection of the mucosal barrier by pathogenic bacteria such as *Pseudomonas aeruginosa*. These pleiotropic activities of the LL-37 peptide likely occur due to its agonistic effect on various membrane receptors. The chemotactic properties of LL-37 involve its interaction with formyl peptide receptor like-1 (FPRL-1) (De Yang et al. 2000). Activation of FPRL-1 by LL-37 has been implicated in immune surveillance against neoplastic transformation, wherein cells of the immune system such as natural killer lymphocytes (NKs) and type 1 CD4^+^ T lymphocytes recognize and destroy cancer cells (Ostrand-Rosenberg 2008). Such tumor-suppressing activity of formyl peptide receptor family after activation with LL-37 peptide was also recently reported for gastric cancer (Prevete et al. 2015). Conversely, the interaction of LL-37 with FPRL-1 has also been implicated in the metastatic progression of ovarian cancer cells, via FPRL-1-mediated recruitment of mesenchymal stromal cells (MSCs) (Coffelt et al. 2009).

The biological actions of LL-37 peptide are also mediated by the activation of purinergic receptor P2X7 and epidermal growth factor receptor (EGFR) (von Haussen et al. 2008). Activation of P2X7 leads directly to increased secretion of proinflammatory cytokines interleukin (IL)-1β and IL-8 (Ferrari et al. 2006; Montreekachon et al. 2011), polymorphisms of which have recently been implicated in some types of malignancies (Gao et al. 2014; Koensgen et al. 2015). LL-37 acts as a physiological immunomodulator that might control the inflammation at infection/injury sites and influences the process of cell regeneration. Furthermore, LL-37 is able to activate insulin-like growth factor-1 receptor (IGF-1R), which results in increased cell proliferation and expression of metastatic phenotype (Girnita et al. 2012).

In addition to its functions in human immunity, cathelicidin plays a role as an inducer of angiogenesis (Koczulla et al. 2003). This notion is supported by an important link between LL-37 and regulation of apoptosis. It is generally accepted that a loss of balance between cell proliferation and cell death is essential in tumor development (Ouyang et al. 2012) and regulation of apoptosis by LL-37 might be involved in pathogenesis of malignant tumors.

LL-37-induced apoptosis explains its antitumor activity in colon cancers and hematologic malignancies (Mader et al. 2009; Ren et al. 2012, 2013). However, LL-37 can also promote tumor growth, depending on the tissue from which the cancer cells originate. Indeed, in various types of cancer a different expression of LL-37 peptide was observed. In ovarian, lung, breast cancer and malignant melanoma cells increase expression was reported (Bals et al. 1998; Coffelt et al. 2008; Heilborn et al. 2005; Kim et al. 2010). In contrast, cells from colon or gastric cancers produce lower amounts of this peptide (Hase et al. 2003; Ren et al. 2012). These observations imply that the actions of LL-37 are tissue-specific (Table 1). In this review, we summarize the role of LL-37 in the development of the most common types of cancer. We emphasize that in many of these, the mechanisms by which LL-37 exerts its pro-tumorigenic or anti-cancer effect have not been fully understood.

## Ovarian Cancer

**Fig. 2:**
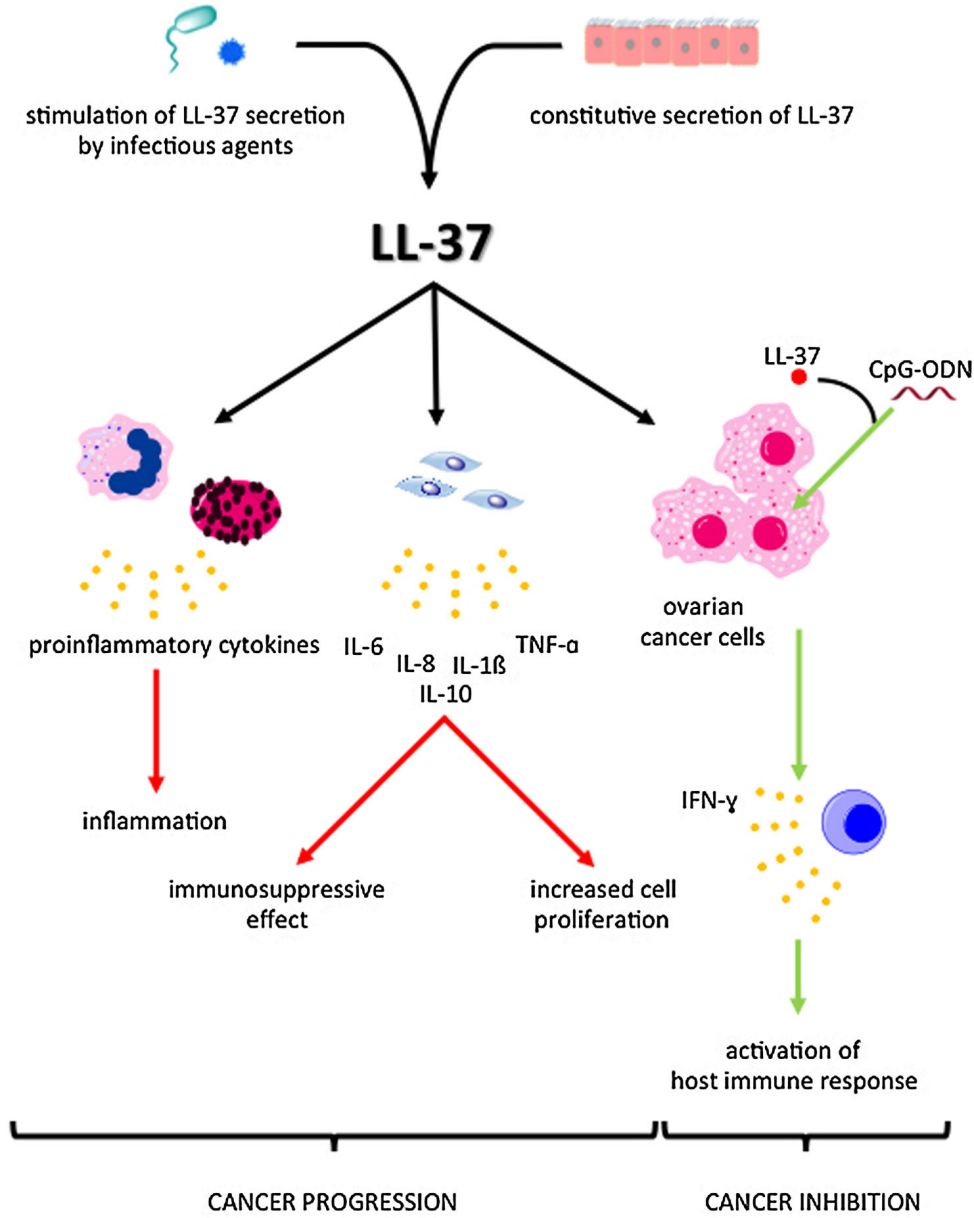
Dual role of LL-37 in ovarian cancer development. *LL-37:* cathelicidin LL-37, *CpG-ODN:* CpG oligodeoxynucleotides, *TNF-α:* tumor necrosis factor α, *IFN-γ:* interferon γ, *IL:* interleukin

Each year, over 200,000 women are diagnosed with ovarian cancer, and although up to 44 % of women survive 5 years after diagnosis, ovarian cancer is the leading cause of death among all gynecological malignances (Muccioli and Benencia 2014; Romero and Bast 2012). A number of studies have shown that proinflammatory factors are involved in ovarian cancer development (Clendenen et al. 2011; Macciò and Madeddu 2012). In particular, tumor necrosis factor (TNF)-α, IL-1β and IL-6 are associated with ovarian tumorigenesis (Clendenen et al. 2011). Additionally, the correlation between increasing C-reactive protein level and risk of ovarian cancer development was described (Clendenen et al. 2011; McSorley et al. 2007). Recent reports have demonstrated increased secretion of LL-37 in ovarian tumors compared to normal ovary cells, implicating the role of LL-37 in ovarian cancer development (Fig. 2) (Coffelt et al. 2008). Based on these results we suggest that measurement of hCAP-18/LL-37 blood level might be proposed as a potential biomarker for ovarian cancer. Interestingly, the increase in concentration of the LL-37 peptide was observed in early stage, i.e. grade I ovarian tumors (Lim et al. 2013). Considering that a significant mortality from ovarian cancer is largely due to late detection, the identification of for ovarian cancer early markers could significantly increase the survival of women affected by this disease.

### Tumorigenic Effect of LL-37 in Ovarian Cancer Tissues Via MSC Recruitment

Several studies indicate that overexpression of LL-37 peptide in ovarian epithelial cells acts as a positive regulator of ovarian cancer progression. LL-37 promotes tumorigenesis through its ability to endorse engraftment of bone marrow-derived, multipotent mesenchymal stromal/stem cells (MSCs) into the tumor stroma, where they contribute to tumor progression and metastasis (Coffelt et al. 2009; Wu et al. 2010c). MSCs also carry a high capacity for the production of a variety of cytokines and growth factors, ultimately inducing immunomodulatory effects on various cells associated with carcinogenesis (Chen et al. 2011; Coffelt et al. 2009; Wu et al. 2010c). LL-37 augments the pro-tumorigenic nature of MSCs by recruiting them to ovarian tumors through FPRL-1 (Coffelt et al. 2009). Neutralization of LL-37 in vivo, using an anti-LL-37 antibody, significantly reduced the engraftment of MSCs into ovarian tumor xenografts developed from OVCAR-3 ovarian cancer cells, resulting in inhibition of tumor growth as well as disruption of the fibrovascular network (Coffelt et al. 2009).

In addition to LL-37, several cytokines, including IL-6, stromal cell-derived factor 1 and prostaglandin E2 (PGE2) were implicated in MSCs recruitment, thus neutralization of LL-37 did not completely block MCSs migration (Coffelt et al. 2009; Touboul et al. 2014). However, LL-37 resulted in a significant number of vascular channels in nude mice. These data indicate that LL-37-mediated recruitment of MSCs can also facilitate ovarian tumor progression through secretion of pro-angiogenic factors. Thus, a significant contributor to the role of LL-37 in ovarian cancer development is its enhancement of MSCs secretion of IL-1β, IL-6, IL-8, IL-10 and TNF-α (and reduction of IL-12 expression). Consistently, in vitro endothelial cell formation by MSCs is enhanced with LL-37 presence with positive effect on tumor growth (Touboul et al. 2014). Finally, MSCs are associated with enhanced tumor aggressiveness by their immunosuppressive effects on NKs or T cells, which reduce the body’s response to the growing tumor (Chen et al. 2011).

### LL-37 Enhances Chemotherapeutic Effects of CpG Oligodeoxynucleotides

Opposing the angiogenic and inflammatory activities of LL-37-mediated recruitment of MSCs, LL-37 has shown synergistic toxicity against ovarian cancer cells in combination with other Toll-like receptor (TLR) ligands such as CpG-ODN (Chuang et al. 2009; Hurtado and Peh 2010). Co-administration of LL-37 and CpG-ODN increases the delivery of CpG-ODN into the endosomal compartments, where it can bind to TLR9 and activate the immune system. This effect might result from the membrane activity of LL-37, as its insertion into the plasma membrane may affect membrane fluidity and bilayer membrane architecture. The combined CpG-ODN/LL-37 therapy, expression of interferon (IFN)-γ, inducing proliferation and activation of natural killer (NK) cells, but not CD4^+^ or CD8^+^ T cells, in the peritoneal cavity, ultimately increase the organism’s natural defenses against the cancer cells (Chuang et al. 2009).

## Lung Cancer

The presence of hCAP-18/LL-37 in epithelial cells of human lungs as well as in mucous cells of the submucosal glands is well studied (Agerberth et al. 2000; Scott et al. 2002; Vandamme et al. 2012). Additionally, LL-37 is secreted into the airway surface fluid, where it serves its most recognized function of defending the conducting airway against pathogenic microorganisms, via its antimicrobial activity against a large spectrum of respiratory pathogens, including respiratory syncytial virus, influenza viruses, *P. aeruginosa,* and *Staphylococcus aureus* (Bals et al. 1998; Barlow et al. 2011; Currie et al. 2013; Dosler and Karaaslan 2014; Travis et al. 2000; Wang 2014). LL-37 also facilitates the regeneration of damaged lung epithelial tissues (Shaykhiev et al. 2005). However, a loss of control of this mitogenic activity might be associated with lung cancer.

### LL-37 is Up-Regulated and Acts as Growth Factor in Lung Cancer

Conducted studies revealed that lung cancer cells express hCAP-18/LL-37 at higher levels. In a large percentage of patients diagnosed with squamous cell cancer and adenocarcinoma, increased levels of cathelicidin were also observed in serum. The same effect was confirmed in both in vivo and in vitro models. Those studies revealed that human cathelicidin stimulates proliferation of bronchial cancer cells and established a positive correlation between the progression of lung cancer and the blood level of LL-37 (von Haussen et al. 2008).

The pro-tumorigenic mechanism of LL-37 appears to involve the activation of EGFR (von Haussen et al. 2008), a tyrosine kinase receptor widely implicated in lung cancer development. Addition of the LL-37 peptide at low concentrations (5 ng/ml) to lung cancer cell lines induced phosphorylation of the EGFR and activation of downstream MAP kinase signaling pathways, leading to enhanced proliferation and growth of anchorage-independent colonies. Lung cancer cell lines stably overexpressing the LL-37 peptide by means of a doxycycline-regulated promoter system also showed faster growth. The mechanism behind these observations may be related to LL-37-mediated activation of a metalloproteinase that cleaves membrane-anchored EGFR ligands (Tjabringa et al. 2003; von Haussen et al. 2008).

### Potential Role of LL-37 in Lung Cancer Development is Link to IL-32 Expression

IL-32, previously known as NK-4, is one of the recently described proinflammatory cytokines produced by immune cells (monocytes, T lymphocytes) and non-immune epithelial and endothelial cells. IL-32 induces the production of several other cytokines and chemokines, including TNF-α, IL-1β, IL-6, IL-8 or IL-18, and activates the NF-κB and p38 MAP kinase pathways (Dinarello and Kim 2006; Felaco et al. 2009; Yousif et al. 2013). These properties, coupled with pro-angiogenic attributes (Nold-Petry et al. 2014) of IL-32, make it an interesting candidate in cancer development. Expression of IL-32, produced during autoimmune disease development including inflammatory bowel disease was found to increase in cervical cancer (Joosten et al. 2006; Lee et al. 2011; Shioya et al. 2007). Expression of IL-32 also rises during *Mycobacterium tuberculosis* infection, and caspase-1/IL-18/IFN-γ pathway governs its production (Netea et al. 2006). Given that *M. tuberculosis* infections are risk factors associated with development of lung cancer, IL-32 was suggested as a possible link between chronic lung infection and lung cancer development (Simonsen et al. 2014). Indeed, recent studies indicate that the expression of IL-32 is significantly altered in lung cancer cells compared to normal cells. It occurs in advanced cancer and exhibits a histology-specific type pattern (Sorrentino and Di Carlo 2009). Expression of IL-32 is also associated with increased lung cancer invasiveness, metastatic ability and poor prognosis (Zeng et al. 2014). In all lung cancer histotypes stromal leukocytes may also account for a considerable expression of IL-32, inside both, IL-32 positive and IL-32 negative tumors. IL-32 expressing leukocytes were identified as CD68^+^ macrophages and CD3^+^ T cells, mainly of CD4^+^ phenotype, both of which are well-known IL-32 producers. IL-32 ability to activate gene transcription factor—NF-κB, was propose to promote lung cancer progression. LL-37 and its shorter derivative IG-19 significantly suppress IL-32-induced production of pro-inflammatory cytokines such as TNF-α and IL-1β. In contrast, LL-37 and IG-19 enhance the production of the anti-inflammatory cytokine IL-1RA. LL-37 and IG-19 suppress IL-32-mediated phosphorylation of Fyn (Y420) Src kinase. However, IL-32-mediated phosphorylation of AKT-1 and MKP-1 was not suppressed. LL-37 and IG-19 alone induce the phosphorylation of MKP-1, which is a known negative regulator of inflammation. Furthermore, the peptides induce the activity of p44/42 mitogen-activated protein kinase (MAPK), which is known to phosphorylate MKP-1. Taking into the account previous studies showing an antagonistic activity of LL-37 against IL-32 in gastric cancer cells (Choi et al. 2014), it might be assumed that similar relationship function during lung cancer development.

## Breast Cancer

LL-37 is an important factor of the innate human defense system of the mammary gland epithelium in human breast (Armogida et al. 2004). However, increased expression of LL-37 was also observed in breast cancer cells, with secreted concentrations correlating to phenotypic tumor severity (Heilborn et al. 2005). Evaluation of hCAP18/LL-37 ability to promote breast cancer development revealed that metastatic potential greatly increases as a result of augmented Heregulin-mediated mitogenic signaling through ErbB2. A modified version of LL-37 competitively inhibited LL-37 induced MAPK phosphorylation and drastically reduced cancer cell colonies induced by LL-37, in addition to inhibiting cancer cell migration (Weber et al. 2009).

## Colon Cancer

Early diagnosis of colon cancer is crucial for therapeutic success, since mortality is high in the late stages of disease—median length of survival is 5–6 months (Van Cutsem and Geboes 2007). In 2012, over 100,000 new cases were diagnosed in the United States alone (Mishra et al. 2013). In most colon cancer cells, a complete loss of hCAP18/LL-37 expression was noted (Ren et al. 2012). Therefore, low levels of LL-37 expression might serve as a biomarker of colon cancer (Lim et al. 2013; Ren et al. 2012).

### LL-37 and Its Derivatives FK-16 and FF/CAP-18 Suppress Colorectal Cancer Cells Growth

**Fig. 3:**
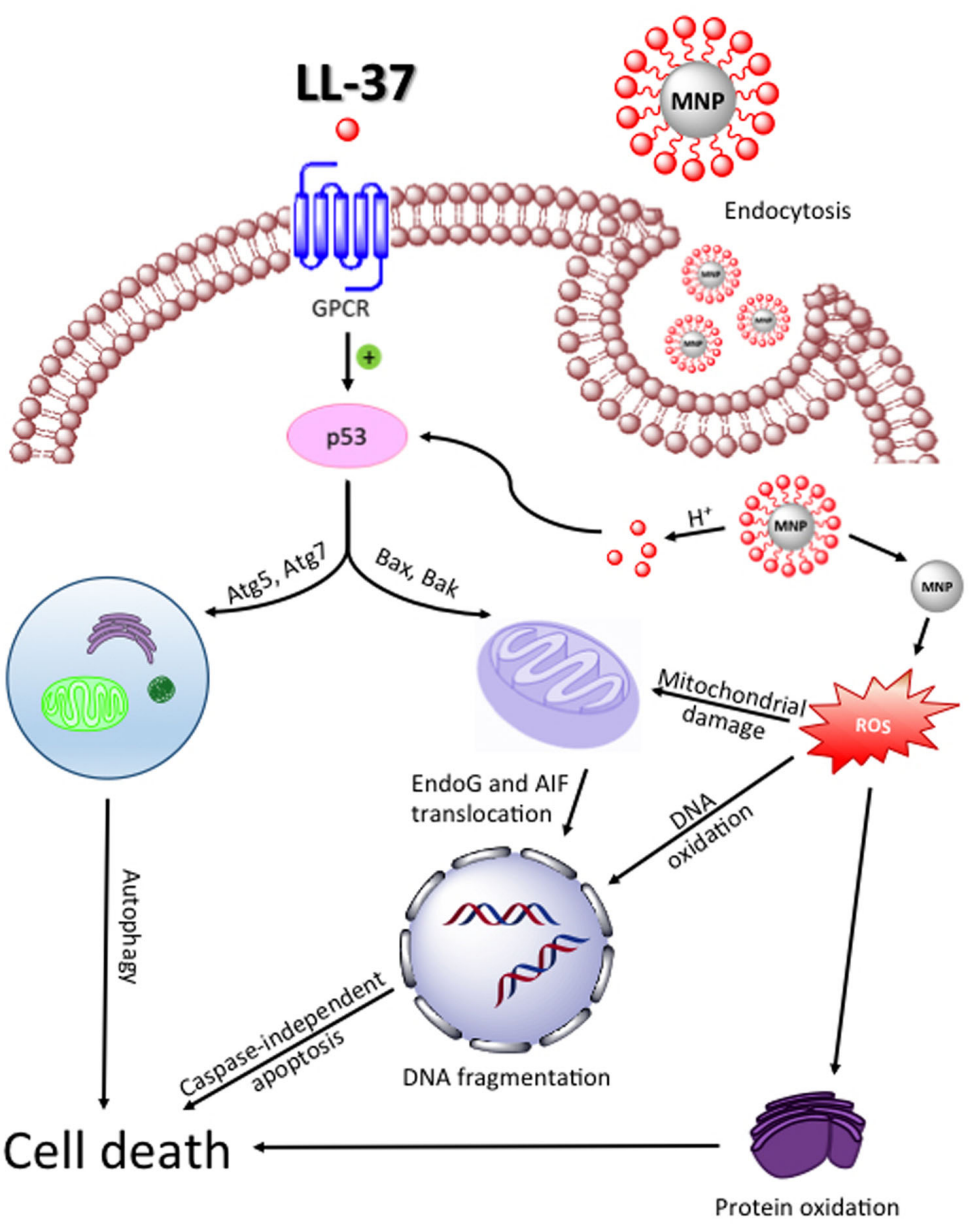
Activation of autophagy and caspase-independent apoptosis pathway and proposed mechanism of action of magnetic nanoparticles functionalized with LL-37 on colon cancer cells. *LL-37:* cathelicidin LL-37, *GPCR:* G-protein coupled receptor, *p53:* tumor protein p53, *Bax:* BCL2-assiociated X protein, *Bak:* Bcl-2 homologous antagonist, *Atg5/7:* autophagy-related protein 5/7, *EndoG:* endonuclease G, *AIF:* apoptosis inducing factor, *MNP:* magnetic nanoparticles, *ROS:* reactive oxygen species

Decreased secretion of LL-37 peptide in colorectal cancer facilitates cancer growth. Interestingly, LL-37 and its fragments, FK-16 (16-residue peptide derived from residues 17–32 of LL-37—discovered and demonstrated to have anti-cancer activity by Li et al. (2006)) and FF/CAP-18 (designed by replacement of glutamic acid and lysine residue with phenylalanine) display anti-proliferative effect against colon cancer cells (Kuroda et al. 2012; Ren et al. 2012, 2013). Pro-apoptotic activity of LL-37 and its derivatives may be enhanced using magnetic nanoparticles (MNPs) as drug delivery systems (Fig. 3). Recent studies revealed, that immobilization of LL-37 on the surface of MNPs significantly increases antitumor activity of LL-37 against colon cancer DLD-1 and HT-29 cell lines (Niemirowicz et al. 2015).

**Table 1:** The mechanism of pro-tumorigenic and anti-cancer activity of LL-37 and alternation of its expression in various types of cancer

Type of cancer	Change in expression of LL-37	Mechanism of pro-tumorigenic/anti-cancer activity of LL-37 in cancer cells	References
Ovarian cancer	↑	Recruitment of MSCs and increasing of their invasiveness and immunosuppressing effect via FPRL-1	Castells et al. (2013), Coffelt et al. (2008, 2009), Cohen et al. (2013)
		Enhancing chemoresistance of cancer cells	
		Increasing of therapeutic effect of CpG-ODN (augmented delivery of CpG-ODN into cancer cells), increasing of proliferation and activation of immune cells	Chuang et al. (2009)
Colon cancer	↓	Activation of cancer cell apoptosis in caspase-independent manner via GPCR-*p53*-*Bcl*-*2/Bax/Bak*-*AIF/EndoG* cascade	Kuroda et al. (2012), Ren et al. (2012, 2013)
		Promotion of cancer cells death via autophagy process	
Gastric cancer	↓	Control of the IL-32γ-induced inflammation by activation of p44/42 MAPK	Choi et al. (2014), Hase et al. (2003), Prevete et al. (2015), Wu et al. (2010b)
		Inhibition of proteasome in gastric cancer tissues and activation of BMP/p52 cascade	
		Inhibition of angiogenesis via FPR1 activation	
Lung cancer	↑	Activation of EGFR and MEK/ERK1/2 signaling pathway	von Haussen et al. (2008)
Breast cancer	↑	Stimulation of ERB-family receptors	Heilborn et al. (2005)
Malignant melanoma	↑	Unstudied	Kim et al. (2010)
Hematological malignancies	↓	Induction apoptosis of cells in caspase-independent manner	An et al. (2005), Mader et al. (2009)
Prostate cancer	↑	Initiation of phosphorylated Erk1/2 and Akt signaling pathway	Hensel et al. (2011)
Open-ended		Inhibition of telomerase enzyme via binding telomeric G-quadruplex	Neidle (2010)
Open-ended		β-Arrestin-1-dependent activation of MAPK/ERK signaling pathway via activation of IGR-1R	Girnita et al. (2012)

LL-37 appears to interfere with colon cancer development by affecting the epithelial-mesenchymal transition and tumor-associated fibroblasts. The mechanism of this effect is related to the cytoskeletal architecture of fibroblasts, which is related to their ability to support division of colon cancer cells. Importantly, animal models suggested that the cathelicidin protein family directly prevented colon cancer growth, as administration of mouse cathelicidin peptide by enema to the area of colon cancer growth significantly reduced the number and area of colonic tumors. Additionally, at the cytoskeletal level, it was observed that cathelicidin preferentially affected tumoral vimentin, but not mouse host fibroblast vimentin. In view of the fact that vimentin production in tumor stroma is linked with short survival time of patients suffering from colon cancer, targeted treatment with cathelicidin appears to be a promising direction (Cheng et al. 2014).

### LL-37 and FK-16 Activate Caspase-Independent Apoptosis in Colon Cancer Cells

In some cancer cells, stimulation of colon cancer cells by LL-37—and its derivative peptides—induces hallmarks of apoptosis, including phosphatidylserine externalization and DNA fragmentation, without activation of caspases (Ren et al. 2012). Induction of apoptosis in caspase-independent manner was verified by analyzing the cleavage of Poly (ADP-ribose) polymerase (Galluzzi et al. 2008). Not only was caspase activity not increased, but even a decrease in the activity of these enzymes was noted, strengthening the hypothesis of caspase-independent mechanism of LL-37 action (Ren et al. 2012). Initiation of apoptosis without activation of caspases is an important observation, considering the latest data indicating the impact of caspase-dependent apoptosis for repopulation of cancer cells (Donato et al. 2014; Huang et al. 2011). Recent studies suggest that activated caspase-3 positively regulates cancer cell proliferation and stimulates tumor growth via increased activation of PGE2—one of the factors that can strongly stimulate growth of surviving cancer cells.

Caspase-independent apoptosis of colon cancer cells is mediated by apoptosis inducing factor (AIF) and endonuclease G (EndoG), involved in DNA degradation. For this activity, the proteins must be translocated from mitochondria, where they normally localized, into the nucleus. LL-37 significantly increases the nuclear level of AIF and EndoG (Ren et al. 2012). Another requirement for caspase-independent apoptosis of cancer cells is increased activity of Bcl-2 and p53 (Arnoult et al. 2003). The expectation that LL-37 would increase the production of pro-apoptotic Bax and Bak and reduce the level of antiapoptotic Bcl-2 was experimentally confirmed, in addition to the observation of the enhanced expression of PUMA, a direct target gene of *p53* in HCT116 cells, after treatment with LL-37 (Ren et al. 2012). It was reported, that viability of colon cancer cells is also reduced in response to treatment with FK-16 (Ren et al. 2013). Here again, a caspase-independent mechanism was inferred, evidenced by DNA fragmentation and phoshatidylserine externalization via *Gi*-coupled GPCR-*p53*-*Bcl*-*2/Bax/Bak*-*AIF/EndoG* cascade. FK-16 actually possessed higher potency against colon cancer cells compared to LL-37, though the viability of LoVo and HCT116 cells was significant reduced by both peptides, suggesting both LL-37 and FK-16 as interesting possibilities for colorectal cancer treatment (Ren et al. 2013).

Finally, the biological activity of LL-37 analogue FF/CAP18 and its influence on the viability of the colon cancer cells line HCT116 has been investigated as well. Interestingly, FF/CAP18 possesses greater growth inhibition capability, when it is compared to LL-37. Detailed analysis of the observed effect again supports the conclusion that decreased survival of cancer cells is mediated by the induction of the caspase-independent apoptotic pathway via the loss of mitochondrial membrane potential. The increased effectiveness of FF/CAP-18 in comparison with the LL-37 is likely attributable to the higher negative charge (Kuroda et al. 2012).

### FK-16 Initiates Cell Death Via Autophagy

With respect to the mechanism of cell toxicity of LL-37 derivatives, one of the key findings was the observation of FK-16-initiated autophagic cell death (autophagocytosis) via increased expression of autophage-related proteins LC3-I, LC3-II, Atg5, and Atg7 (Ren et al. 2013). Autophagy may play a dual role in cancer development, either inhibiting or promoting tumor growth (Wu et al. 2012). For example, the key tumor suppressor protein p53 positively regulates autophagy in cancer cells (Levine and Abrams 2008; Zeng et al. 2007). The study showed that the FK-16 fragment induces cell death by both caspase-independent apoptosis and autophagy. Particularly interesting is the observation linking both these processes. It was found that the abolition of autophagy results in increasing cell death via apoptosis and vice versa. This dual, mutually dependent mechanism of inducing cell death is essential for anti-cancer activity of FK-16 (Ren et al. 2013). The activation of p53 in colon cancer cells observed under LL-37 or FK-16 treatment underline the potential of this pathway as a new approach towards colon cancer therapy.

## Gastric Cancer

Many factors are involved in development of gastric cancer. Among them, *Helicobacter pylori* plays a key role in stomach carcinogenesis, as evidenced by epidemiological and clinical studies (Kim et al. 2014; Wu et al. 2010b). However, the newest survival analysis conducted in 2014 revealed that *H. pylori* infection as a prognostic factor for gastric cancer patient is not clear (Kim et al. 2014). There is an intimate relationship between cancer and inflammation, and *H. pylori* infection induces inflammation and intensifies secretion of pro-inflammatory cytokines. Inflammation associated with chronic gastritis was linked with neoplastic formation (Alzahrani et al. 2014). The fact that hCAP-18/LL-37 is also released during inflammatory processes suggests a possible link between these peptides and gastric cancer.

### LL-37 is Up-Regulated during *H. pylori* Infection, but is Down-Regulated in Gastric Cancer Tissues

Based on the research carried out by Choi et al. (2014) it was established that LL-37 acts as anti-cancer agent during *H. pylori* induced inflammation and cancer development. hCAP-18/LL-37 is constitutively secreted by surface epithelial cells and epithelial cells in the fundic glands, and its expression is enhanced in the case of *H. pylori* infection, where it exerts protective and bactericidal effects. In contrast, the level of LL-37 in various types of gastric cancers is significantly reduced (Hase et al. 2003; Wu et al. 2010b, c). This discovery was the starting point of the hypothesis that LL-37 possesses antitumor properties.

### LL-37-Mediated Inhibition of Proteasome Limits Gastric Cancer Development

LL-37 in gastric cancer cells acts as cancer-suppressing agent due to its ability to inhibit the proteasome in gastric tumor tissues (Wu et al. 2010b). The proteasome is the main system linked with intracellular protein degradation and its connection with cell proliferation, apoptosis, and stabilization of cell cycle has made it an attractive target for cancer therapy (Krętowski et al. 2015; Richardson et al. 2005; Steg et al. 2014; Wu et al. 2010a; Yerlikaya et al. 2015). In gastric cancer cells, human cathelicidin inhibited the proteasome, promoting signaling through the bone morphogenic protein (BMP) pathway. This effect leads to increased expression of BMP4, up-regulation of cycline-dependent kinase inhibitor p21^WafI/ClipI^, and down-regulation of cyclin E_2_. Considering that cyclin E_2_ and p21^WafI/ClipI^ are important factors in regulating the cell cycle (Bartek and Lukas 2001), alternations in their activities likely play a key role in the antitumor action of LL-37 via induction of G_0_/G_1_-phase cell cycle arrest. Interestingly, enlarged concentration of LL-37 is not necessary to achieve the antitumor effect in the cancer cells. It was revealed that LL-37 inhibits cells proliferation at the physiological levels (Wu et al. 2010b). In light of these and previous reports, down-regulation of LL-37 appears to be an important checkpoint in gastric cancer development.

### LL-37 and IG-19 Activate p44/42 MAPK and Control IL-32-Induced Inflammation

As in the case of lung cancer, pro-tumorigenic properties of IL-32 were also confirmed for gastric cancer. In the context of this malignancy, up-regulation of IL-32 is not only associated with *H. pylori* infection, but also can be used as prognostic marker for gastric cancer patients since its expression is increased in human gastritis and gastric cancer cells, compared to normal cells (Ishigami et al. 2013; Peng et al. 2014; Sakitani et al. 2012). Recent studies revealed that LL-37 and its derivative IG-19 (amino acids rest 13–31) play an important role in controlling IL-32γ-induced inflammation (Choi et al. 2014). Both peptides reduced the levels of IL-32, leading to reduce production of proinflammatory cytokines such as TNF-α or IL-6. This effect was observed both in peripheral blood-derived mononuclear cells and macrophages. Interestingly, the expression of the anti-inflammatory cytokine IL-1 receptor antagonist (IL-1RA) was not affected by either LL-37 or IG19, suggesting that LL-37 attenuates inflammation in the mucous membranes of the stomach, potentially also attenuating tumor growth within these tissues. The mechanism of this anti-inflammatory effect was associated with activation of p44/42 MAPK and the subsequent phosphorylation of MKP-1 (S359) (Choi et al. 2014). This observation is consistent with other reports that dual phosphatase MKP-1 plays a general role in reducing inflammation in gastric tissues (Korhonen and Moilanen 2014). It should be emphasized that LL-37 and IG-19 do not negatively affect the production of chemokines (chemotactic cytokines, whose major role is act as a chemoattractant to control the migration of cells), which play an important role in immune response to infection (Choi et al. 2014).

## LL-37 in Other Malignant Tumors

### Malignant Melanoma

LL-37 is secreted by human skin cells in increased amounts in response to the inflammation and infection, acting as a protective factor to reduce the possibility of infection and complications associated with the dysfunction of skin homeostasis (Frohm et al. 1997). On the other hand, immunohistochemical analysis of LL-37 expression in skin tumors revealed that there might be a relationship between the level of hCAP-18/LL-37 and the development of cancerous conditions in these cells. Several in vitro studies indicate that secretion of LL-37 in malignant melanoma cells is significantly increased compared to normal skin and hematological malignant cell lines, suggesting the possibility that LL-37 acts as a growth factor for skin tumor cells and enhances cancer development. However, the increased expression of LL-37 cannot be used as prognostic factor for patients diagnosed with malignant melanoma, since no difference in the expression of LL-37 between clinical subtypes of malignant melanoma was found (Kim et al. 2010). The mechanism by which the LL-37 would exhibit the pro-tumorigenic properties in malignant melanoma has not been thoroughly investigated. However, there are some reports on the basis of which we can hypothesize the possible link between up-regulated level of LL-37 and melanoma cancer cell growth. In a recent study, a specific role of TLR-4 in the process of melanoma cells growth was reported. Not only was TLR-4 secreted in considerable amounts in human melanomas, but it was shown to be important for cancer cell growth and migration (Takazawa et al. 2014). This result is consistent with previous reports about the involvement of TLR-4 in cancer development (Mai et al. 2013). Since LL-37 is one the endogenous agonists for TLRs-family, including TLR-4 (Mookherjee et al. 2006), we suspect that possible interactions between LL-37 and TLR-4 may impact neoplastic formation.

### Hematologic Malignancies: Lymphoma and Leukemia

In contrast to ovarian, breast or lung cancer, where strong evidence of pro-tumorigenic effects of LL-37 was described, in hematologic malignancies, LL-37 might be considered as a therapeutic agent. For example, LL-37 activates apoptosis in a malignant human T cell line (Jurkat). This effect requires an increase of intracellular Ca^2+^ concentration and activation of calpains. LL-37 induces cell death in caspase-independent manner via loss mitochondrial transmembrane potential, connected to translocation of Bax to mitochondria and subsequent AIF transport to the nucleus (Mader et al. 2009). These results are particularly intriguing in light of research showing that the expression level of LL-37 in patients with leukemia is significantly reduced (An et al. 2005), which may affect not only tumor progression, but also resistance to infections in patients diagnosed with leukemia.

### Prostate Cancer

Pro-tumorigenic properties of LL-37 were also described in development of prostate cancer. In both mouse models and human prostate cancer cells, the expression of LL-37 is altered (Hensel et al. 2011). The level of LL-37 is not only augmented compared with healthy cells, but are also associated on the severity of the disease and endocrine nature of the tumor. Those properties suggest that the level of this peptide may be a useful prognostic factor, since expression of LL-37 increases in parallel with the Gleason scale, classification system for prostate cancer based on an assessment of the histological structure of tumor growth. Additionally, androgen-independent cells secrete more LL-37 than androgen-dependent cells, which can be useful in disease diagnosis. Finally, LL-37 enhances invasiveness of tumor cells via phosphorylated Erk1/2 and Akt signaling pathway (Hensel et al. 2011).

## Other Properties of LL-37 Linked with the Development of Tumors

### LL-37 Affects Telomerase Activity

LL-37 acts as an activator of apoptosis, although the exact mechanism by which LL-37 induces apoptosis is not fully understood. In addition to the cascade based on Bax and AIF described above, a recent study indicates that LL-37 is a binder of the telomeric G-quadruplex (Jana et al. 2013). G-quadruplexes are four-stranded DNA structures that inhibit telomerase activity and play a key role in the conservation of telomere length. Given that overexpression of telomerase is observed in 85–90 % of human cancers, the possibility of inactivating this activity has become an extremely attractive target for novel anti-cancer treatments (Folini et al. 2009; Neidle 2010). LL-37 appears to stabilize G-quadruplexes and thereby reduces telomerase activity in cancer cells (Jana et al. 2013).

### LL-37 Activates IGF-1R

Despite the knowledge of multiple signaling pathways through which the LL-37 may enhance or inhibit the development of cancer, new mechanisms of action of the LL-37 peptide are being discovered. For example, in 2012 LL-37 was found to interact with IGF-1R, one of the most widely overexpressed receptors in various types of human cancers (Girnita et al. 2012). IGF-1R appears to play a key role in the control of cell proliferation and metastasic potential (Khandwala et al. 2000). Various experiments show that LL-37 might act as a partial agonist for IGF-1R, triggering β-arrestin-1-dependent activation of MAPK/ERK, without affecting PI3K/Akt activity (Girnita et al. 2012).

## Conclusions

**Fig. 4:**
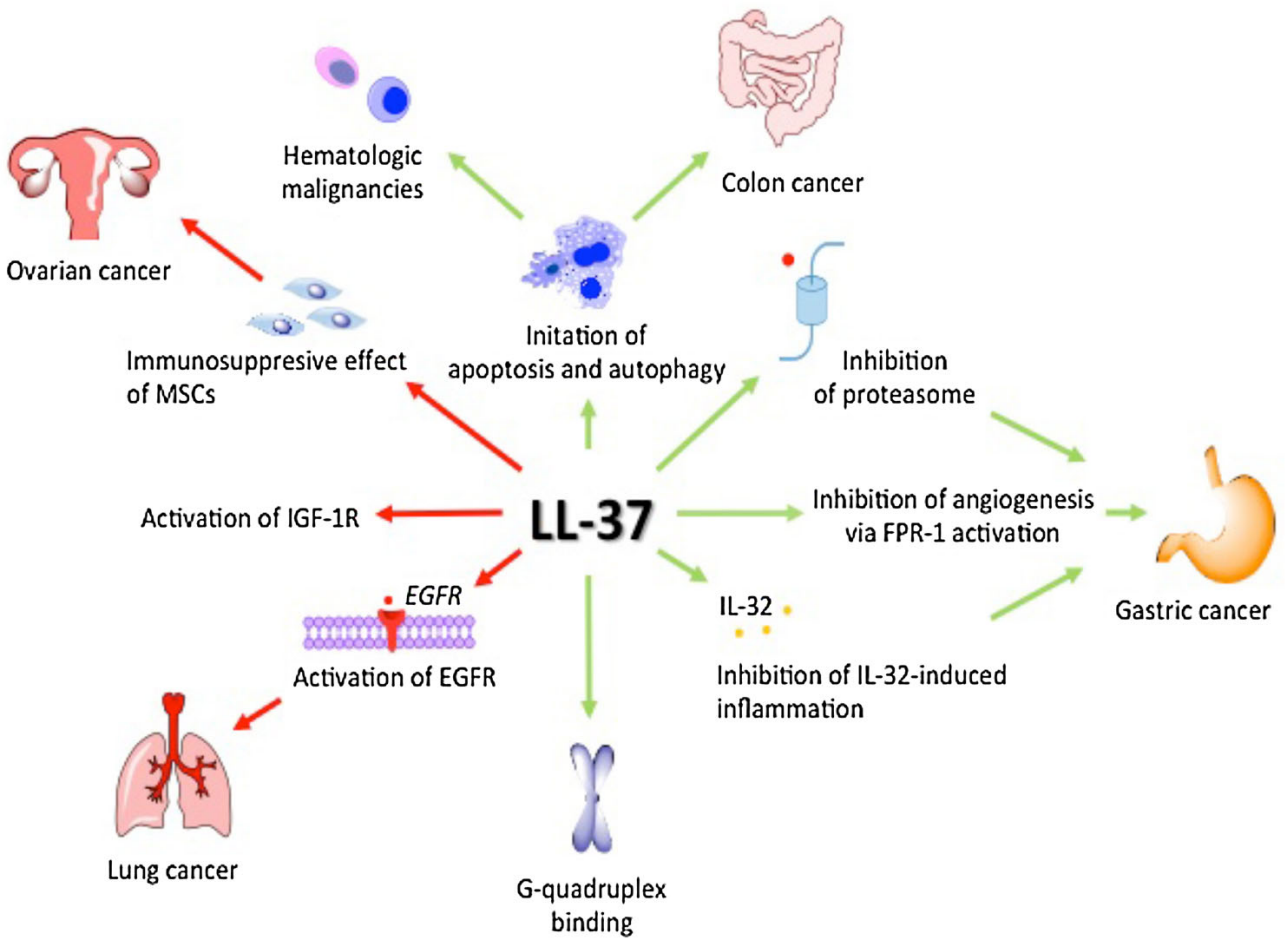
LL-37 effect on cancer development. Green arrows indicate positive effect on cancer cells growth and red arrows indicate pro-tumorigenic properties of LL-37. *LL-37:* cathelicidin LL-37, *MSCs:* mesenchymal stromal cells, *IGF-1R:* insulin-like growth factor 1 receptor, *EGFR:* epidermal growth factor receptor, *FPR-1:* formyl peptide receptor

The research results summarized above clearly indicate that the role of LL-37 in tumor development is tissue-specific (Fig. 4). Depending on tumor biology, LL-37 may act either as a pro-tumorigenic or anti-cancer agent. Moreover, a central issue is the change in the expression of LL-37 in a given tumor compared to healthy tissue of similar origin. Unfortunately, at this point it is not possible to make an indisputable judgment on the effect of LL-37 on cancer development. In 2010, the relationship of LL-37 and cancer was first reviewed (Wu et al. 2010c). Five years later, the same picture remains. Further studies on the biological activity of LL-37 are needed to ultimately define the role of this important peptide and potential therapeutic in carcinogenesis.
